# Bladder Carcinoma Data with Clinical Risk Factors and Molecular Markers: A Cluster Analysis

**DOI:** 10.1155/2015/168682

**Published:** 2015-03-19

**Authors:** Enrique Redondo-Gonzalez, Leandro Nunes de Castro, Jesús Moreno-Sierra, María Luisa Maestro de las Casas, Vicente Vera-Gonzalez, Daniel Gomes Ferrari, Juan Manuel Corchado

**Affiliations:** ^1^Urology Department, Hospital Clinico San Carlos, Complutense University, Instituto de Investigacion Sanitaria San Carlos (IdISSC), 28040 Madrid, Spain; ^2^Natural Computing Laboratory (LCoN), Mackenzie Presbyterian University, 01302-000 São Paulo, SP, Brazil; ^3^Clinical Analysis Department, Hospital Clinico Universitario San Carlos, 28040 Madrid, Spain; ^4^Odontology School, Complutense University, 28040 Madrid, Spain; ^5^Biomedical Research Institute of Salamanca/BISITE Research Group, University of Salamanca, Edificio I+D+i, 37008 Salamanca, Spain

## Abstract

Bladder cancer occurs in the epithelial lining of the urinary bladder and is amongst the most common types of cancer in humans, killing thousands of people a year. This paper is based on the hypothesis that the use of clinical and histopathological data together with information about the concentration of various molecular markers in patients is useful for the prediction of outcomes and the design of treatments of *nonmuscle invasive bladder carcinoma* (NMIBC). A population of 45 patients with a new diagnosis of NMIBC was selected. Patients with *benign prostatic hyperplasia* (BPH), *muscle invasive bladder carcinoma* (MIBC), *carcinoma in situ* (CIS), and NMIBC recurrent tumors were not included due to their different clinical behavior. Clinical history was obtained by means of anamnesis and physical examination, and preoperative imaging and urine cytology were carried out for all patients. Then, patients underwent conventional *transurethral resection* (TURBT) and some proteomic analyses quantified the biomarkers (p53, neu, and EGFR). A postoperative follow-up was performed to detect relapse and progression. Clusterings were performed to find groups with clinical, molecular markers, histopathological prognostic factors, and statistics about recurrence, progression, and overall survival of patients with NMIBC. Four groups were found according to tumor sizes, risk of relapse or progression, and biological behavior. Outlier patients were also detected and categorized according to their clinical characters and biological behavior.

## 1. Introduction

*Bladder cancer* (BC) is one of the most frequently occurring tumors worldwide [1]. Most BCs are* transitional cell carcinomas* (TCC), that is, a cancer that begins in cells that normally make up the inner lining of the bladder. TCC, also known as urothelial carcinoma, is the most common type of bladder cancer. The cancer starts in cells, called transitional cells, in the bladder lining (urothelium).

Bladder cancer is staged according to the degree of tumor invasion into the bladder wall. Carcinoma* in situ* (stage Tis) and stages Ta and T1 are grouped as* nonmuscle invasive bladder cancers* (NMIBC) because they are restricted to the inner epithelial lining of the bladder and do not involve the muscle wall. Of the NMIBC, stage Ta tumors are confined to the mucosa, whereas stage T1 tumors invade the lamina propria. T1 tumors are regarded as being more aggressive than Ta tumors.* Muscle invasive bladder cancer* (MIBC) may extend into the muscle (stage T2), the perivesical fat layer beyond the muscle (stage T3), and adjacent organs (T4). Metastatic tumors involve lymph nodes (N1–3) or distant organs (M1).

Approximately 75% of patients with TCC present a disease at a noninvasive stage that involves only the inner lining of the bladder [2]. The remaining 25% of newly diagnosed bladder cancers are MIBC and have a higher risk of cancer-specific mortality [3] with the need of aggressive radical surgery or radiotherapy, with or without chemotherapy.

The cellular morphology of TCC is graded according to the grading of cellular differentiation. The grading consists of well-differentiated (grade 1), moderately differentiated (grade 2), and poorly differentiated (grade 3) tumors. Grading of cell morphology in NMIBC is important for establishing prognosis because grade 3 tumors are the most aggressive and the most likely to become invasive.

NMIBC is a heterogeneous group of tumors. Between 30% and 90% will relapse within 5 years. One group (70%) will have a good survival rate but a high risk of recurrence with the same degree of clinical aggressiveness and a global survival at 5 years greater than 80% [4]. A minor but not insignificant proportion of patients (30%) [4, 5] have a high risk of progression with a severe worsening of the prognosis and therapeutic options [6]. The main treatment of NMIBC consists of* transurethral resection* (TURBT) followed in the majority of the cases by intravesical instillations of chemotherapeutic agents or immunotherapy.

The heterogeneity of NMIBC in terms of both histological origin and clinical behavior means that clinical parameters such as tumor grade and stage are not yet enough to accurately predict biological behavior or to guide treatment reliably. Although these parameters provide a certain degree of tumor biological potential, a significant degree of tumor heterogeneity remains even within prognostic subgroups. The need for accurate diagnosis, continuous surveillance, and possible repeated treatments and the need to anticipate which NMIBC will progress into an invasive disease make BC one of the most expensive tumors in terms of total medical care expenditures [7] with an estimated cost of US$96,000 to US$187,000 per patient from diagnosis to death in the United States [7]. Accordingly, the major goals in treating patients with NMIBC are to prevent the high number of recurrences and to prevent muscle invasive progression. A more individually tailored follow-up scheme for NMIBC patients depending on their risk profile would help to reduce patient burden and costs. With these aims, new tools to aid diagnosis, assess prognosis, identify optimal treatment, and monitor progression of NMIBC are urgently required.

The unprecedented progress on clinical prognostic accuracy with the emergence of risk calculators, artificial neural networks, and cancer genetics are rapidly affecting the clinical management of solid tumors. Some of them are now an integral part of routine clinical management for patients with lung, colon, and breast cancer. In sharp contrast, molecular biomarkers have been largely excluded from current management algorithms for urologic malignancies. Presently, risk associations are beginning to be included in management algorithms of NMIBC [8], but risk groups and validated prognostic molecular biomarkers that can help clinicians to identify patients in need of early, aggressive management are lacking.

Hierarchical clustering (HC) applied to structured databases is used as an aid to represent medical domain knowledge substructures to simplify the generation process of the databases through clustering. As a result, it is possible to identify interesting relationships and patterns among the data and represent them in the form of rules.

Based on this background there is a belief of the usefulness to employ a prior database used in several studies of our research group [9–13], which includes traditional risk factors, risk groups, and some molecular markers, to perform a cluster analysis to try to discover nonevident patterns in the dataset.

The paper is organized as follows.  Section 2 presents the research hypotheses and goals of the paper.  Section 3 describes the bladder cancer, from epidemiology to etiology, and prognostic factors.  Section 4 presents the population investigated and the clinical methodology used to obtain the data. The hierarchical clustering analysis of the data is presented and discussed in Section 5. The paper is concluded in Section 6 with some considerations and perspectives for future research.

## 2. Research Hypotheses and Goals

The research hypothesis is that a combined molecular and histopathological analysis of NMIBC might be related with predicting outcomes and designing treatments of NMIBC. There are three main goals with this research:

- to find the intrinsic grouping in a set of data with clinical, molecular markers and statistics about recurrence, progression, and overall survival of patients with NMIBC;
- to develop a knowledge discovery in databases (KDD) approach for discovering possible relationships between the concentration of different molecular markers and clinical and histopathological prognostic factors of NMIBC;
- to investigate if a combined clinical and molecular classification of NMIBC based on a developmental biology approach can provide additional prognostic information by using a hierarchical clustering exploratory data analysis.

## 3. Bladder Cancer

### 3.1. Epidemiology of BC

BC is the most common malignancy of the urinary tract, the 7th most common cancer in men and the 17th in women [14]. The worldwide age-standardized incidence rate is 9 per 100,000 for men and 2 per 100,000 for women (2008 data) [15].

In the European Union (EU), the age-standardized incidence rate is 27 per 100,000 for men and 6 per 100,000 for women [1]. The incidence of BC varies between regions and countries; in Europe, the highest age-standardized incidence rate has been reported in Spain (41.5 in men and 4.8 in women) and the lowest in Finland (18.1 in men and 4.3 in women) [15]. 

Worldwide age-standardized mortality rate is 3 for men versus 1 per 100,000 for women. In the EU, the age-standardized mortality rate is 8 for men and 3 per 100,000 for women, respectively [1]. In 2008, BC was the eighth most common cause of cancer-specific mortality in Europe [15].

The incidence of BC has decreased in some areas, possibly reflecting the decreased impact of causing agents, mainly smoking and occupational exposure [16]. Mortality from BC has also decreased, possibly reflecting an increased standard of care [17].

### 3.2. Etiology of BC

Tobacco smoking is the most important risk factor for BC, accounting for approximately 50% of the cases [3, 18], because tobacco smoke contains aromatic amines and polycyclic aromatic hydrocarbons, which are renally excreted. Cigarette smokers have a two- to fourfold increased risk of bladder cancer compared with nonsmokers [19], and the risk increases with increasing intensity and duration of smoking [20]. On cessation of smoking, the risk of bladder cancer falls >30% after 1–4 years and by >60% after 25 years but never returns to the risk level of nonsmokers [1].

Occupational exposure to aromatic amines, polycyclic aromatic hydrocarbons, and chlorinated hydrocarbons is the second most important risk factor for BC, accounting for about 10% of all cases. This type of occupational exposure occurs mainly in industrial plants processing paint, dye, metal, and petroleum products [3, 21, 22].

Although the significance of the amount of fluid intake is uncertain, the chlorination of drinking water and subsequent levels of trihalomethanes are potentially carcinogenic, while exposure to arsenic in drinking water increases the risk [3]. The association between personal hair dye use and risk remains uncertain; an increased risk has been suggested in users of permanent hair dyes with an NAT2 slow acetylation phenotype [23, 24]. The impact of diet and environmental pollution is less evident. 

Exposure to ionizing radiation is connected with increased risk. It is suggested that cyclophosphamide and pioglitazone are weakly associated with BC risk [3]. Schistosomiasis, a chronic endemic cystitis, based on recurrent infection with a parasitic trematode, is a cause of BC [3].

Finally, there is increased evidence that genetic predisposition may influence the incidence of TCC of the bladder [3], especially via its impact on susceptibility to other risk factors [3, 25].

### 3.3. Prognostic Factors (PF) of NMIBC

As previously seen, the NMIBC is a heterogeneous group of tumors whose prognosis and therapeutic indications are very difficult to establish at the diagnosis time. Although TURBT is an essential diagnostic tool and an effective treatment for bladder cancer, 45% of patients will have tumor recurrence within 12 months of TURBT alone. Tumor recurrence can be attributed to a combination of missed tumors, incomplete, initial resection, reimplantation of tumor cells after resection, and tumor occurrence in high risk urothelium. Several factors influence the recurrence rate, for instance, clinical and pathological results, applied treatments, and diagnostics.

There are two fundamental risks attributed to NMIBC: the risk of recurrence without worsening the grade or stage and the risk of progression to MIBC. So, according to this behavior, basically, NMIBC can be classified into three groups of patients. A minority of patients (20–30%) have a relatively benign type of TCC with a low recurrence rate. These low risk tumors do not show progression. The largest group of patients includes those who frequently develop a NMIBC recurrence but seldom experience progression. A third, small group of patients, includes those who have a relatively aggressive non-muscle invasive tumor at presentation. Despite maximum treatment, up to 45% of these patients will develop MIBC. The desire to predict which NMIBC will become MIBC and will develop disseminated disease has stimulated the study of factors with possible prognostic value; these are called prognostic factors (PF).

#### 3.3.1. Clinical PF

The current clinicopathology-based prognostic approaches for predicting recurrence and progression in NMIBC divides the factors into three groups: PF based on clinical; endoscopic; and pathological findings [26–33].

Prognostic factors are based on clinical findings:

- primary or recurrence,
- prior recurrence rate,
- use of intravesical therapy.

PF are based on endoscopic findings:

- number of tumors,
- tumor size.

PF are based on pathologic findings:

- tumor grade,
- tumor stage,
- association with* carcinoma in situ* (CIS).

In our database we selected only primary tumors with no concomitant CIS. Previously recurrent tumors were excluded because of their molecular markers and their natural history could be altered due to the previous use of intravesical chemo- or immunotherapy, usually employed in this kind of tumors. In the same way, concomitant CIS patients were excluded because CIS has a clearly different molecular developmental pathway [34, 35] and a clearly worse prognosis.

Several authors have tried to classify NMIBC risk groups by trying to predict the possible evolution, in order to design strategies for treatment and monitoring.

Parmar et al. [26] established 3 different groups of risk of recurrence: Group 1 (single tumor and negative cystoscopy at 3rd month); Group 2 (multiple tumor, or positive cystoscopy at 3rd month); and Group 3 (multiple tumor and positive cystoscopy at 3rd month). The percentage of patients free of recurrence at 2 years was 74% in Group 1, 44% in Group 2, and 21% in Group 3. In this classification, interesting for its simplicity, the introduction of positive cystoscopy at 3rd month as a risk factor provides a high degree of differentiation of tumor recurrence; however, it is not suitable to assess the progression or tumor mortality, which was not accounted for by this author.

Fradet [36] studying 382 patients with initial NMIBC showed that the main PF for recurrence in their series were tumor multiplicity, size, stage, and tumor grade, defining what they called* adverse tumor characteristics* (ATC). With this classification, recurrence and progression at 1 year were, respectively, 21 and 0% in the low risk group, 36 and 1% in the intermediate risk group, and 66 and 9% in the high risk group. CCAFU [37] also classified the NMIBC into three categories according to progression risk (low risk groups, intermediate, and high).

When using these risk groups, however, no distinction is usually drawn between the risk of disease recurrence and disease progression. Although prognostic factors may indicate a high risk of recurrence, the risk of progression might still be low, while other tumors might have a high risk of both recurrence and progression. 

In order to predict separately the short- and long-term risks of disease recurrence and progression in individual patients, the group of Millán-Rodríguez et al. [38] has also designed its own risk classification based on the PF NMIBC discussed above, with the novelty of having each group assigned a specific weight PF recurrence, progression and mortality, stratifying the different variables depending on the degree and the association of Kish. Accordingly, and analyzing 1,529 patients with NMIBC, they established three groups with different risks of recurrence, progression and mortality. Risk groups were classified as low (grade 1 stage Ta disease and a single grade 1 stage T1 tumor), intermediate (multiple grade 1 stage T1 tumors, grade 2 stage Ta disease, or a single grade 2 stage T1 tumor), and high (multiple grade 2 stage T1 tumors, grade 3 stages Ta or T1 disease, and any stage disease associated with CIS), with significant differences on recurrence, progression, and overall survival among the 3 groups. Low and intermediate risk patients showed 37% and 45% risk of recurrence, respectively, without significant risk for progression or death from bladder cancer. By contrast, in the high risk category the incidence of recurrence, progression, and mortality was 54%, 15%, and 9.5%, respectively.

More recently, the European Organization for Research and Treatment of Cancer (EORTC), Genito-Urinary Cancer Group (GUCG), developed a scoring system and risk tables [8] based on the six most significant clinical and pathological factors:

- number of tumors;
- tumor size;
- prior recurrence rate;
- T category;
- presence of concurrent CIS;
- tumor grade.

The basis for the EORTC risk tables was a combined analysis of individual patient data from 2596 NMIBC patients included in seven randomized EORTC trials [8]. A simple scoring system was derived based on six clinical and pathological factors (number of tumors, tumor size, prior recurrence rate, T stage, presence of concomitant CIS, and tumor grade). Based on available prognostic factors and in particular data from the EORTC risk tables, the EAU Guidelines Panel recommends stratification of patients into three risk groups that will facilitate treatment recommendations.

The prognostic value of the EORTC scoring system has been confirmed by data from the Clube Urológico Español de Tratamiento Oncológico (CUETO) patients treated with BCG and by long-term follow-up in an independent patient population (125,126). The CUETO risk calculator is available at http://www.aeu.es/Cueto.html [39, 40].

For our database, we used a modification of the risk groups classifications proposed by Parmar et al. [26] and Millán-Rodríguez et al. [38], grouping low and intermediate risk groups into the same risk group, trying to avoid the data dispersion, because of the small number of patients in each group and the small prognostic differences between low and intermediate risk groups.

#### 3.3.2. Molecular PF

With increasing understanding of the cellular mechanisms underlying the development of molecular pathways involved in urothelial oncogenesis, some molecular prognostic factors are being proposed to identify patients in need of surveillance and aggressive treatment.

Originally defined to represent the analysis of the entire protein component of a cell or tissue, proteomics now encompasses the study of expressed proteins, including identification and elucidation of the structure-function relationship under healthy conditions and disease conditions, such as in cancer. In combination with genomics, proteomics can provide a holistic understanding of the biology underlying disease processes.

Cancer proteomics encompasses the identification and quantitative analysis of differentially expressed proteins relative to healthy tissue counterparts at different stages of disease, from preneoplasia to neoplasia. Expression analysis directly at the protein level is necessary to unravel the critical changes that occur as part of disease pathogenesis. This is because proteins are often expressed at concentrations and forms that cannot be predicted from mRNA analysis [41].

Many molecular markers have been studied in NMIBC [42], including deletion or expression of mutated forms of the tumor-suppressor genes, p53 and retinoblastoma, and expression of the different products of the tyrosine kinase receptor (TKR) family.

The epidermal growth factor receptor (EGFR) is a member of the TKR family, a group of receptors which are all encoded by the c-*erb*B oncogenes. There are four known c-*erb*B oncogenes whose transcription produces a variety of protein products that play a physiological role in coordinated cell growth and tissue repair. Pathological expression of these proto-oncogenes is associated with the loss of coordination of cell growth that typifies malignancy.

A series of studies have indicated the potential prognostic value of evaluating expression levels of TKR genes such as* FGFR3*,* EGFR*,* ERBB2* (*HER/neu*), and* ERBB3* in patients with NMIBC and muscle invasive bladder cancer (MIBC) [34, 43, 44].

Overexpression of EGFR in bladder cancer has been widely reported [45–48] and several studies have shown EGFR positivity to be associated with high tumor stage, tumor progression, and poor clinical outcome [46, 48, 49]. The mechanism by which EGFR expression is associated with poor prognosis is not entirely clear, although there is some evidence linking EGFR stimulated activation of activator protein-1 transcription factor with induction of matrix metalloproteinase activity [50].

The* HER2/neu* gene encodes a glycoprotein with intrinsic tyrosine kinase activity, another member of the family TKR. The HER2/neu encoded protein molecule occupies a critical position in the biochemical pathways responsible for the transduction of mitogenic signals from a variety of growth factor receptors. In addition to its role in regulating normal cellular proliferation, overexpression of the* HER2/neu* gene appears to play a role in neoplastic cell growth [51].

The incidence of overexpression of HER2/neu in bladder cancer is one of the highest among all human malignancies, ranging from 9% to 34% of the cancers tested [52–55]. In transitional bladder cell carcinoma, it was found that HER2 is overexpressed with a greater frequency in higher grades (40%) and stages (38%) than lower grades (0%) and stages (8%) [56]. Several studies have suggested a negative prognostic role for HER/neu amplification or overexpression in MIBC [57–60]. Using multivariate analysis, Bolenz et al. [55] found that patients harboring tumors with HER/neu overexpression were twice as likely to experience recurrence and to die from their cancer, compared to patients with HER/neu-negative tumors.

A subset of high grade NMIBCs contains HER2 amplification and is associated with markedly aggressive behavior [61]. The results obtained by quantitative methods in other studies showed HER2/neu oncoprotein to be more significantly expressed in the malignant group compared to the benign and normal groups [54], and they concluded that the quantitative assessment of HER2/neu expression in malignant tumors aided by other proliferation markers such as synthetic phase fraction (SPF), DNA index (DI), and ploidy is useful in selecting patients for more aggressive treatment or for predicting outcome.

TP53 tumor suppressor gene is considered to play a significant role in carcinogenesis. Mutations in the TP53 are the most frequent genetic abnormalities encountered in human malignancies, including urinary bladder carcinoma [62]. It has already been established that the half-life of a mutated p53 protein is considerably longer than that of the wild-type p53 protein [63]. The accumulation of the mutated p53 protein in the nuclei of the malignant cell is the main reason for increased detection level by immunohistological methods, including immunofluorescence.

Many previous studies have established that both p53 gene mutations and immunohistochemically detected p53 expression are independent prognostic biomarkers in CCT, indicating that p53 stabilization not encoded by mutant gene could also produce aberrant downstream signaling pathways, with a central role in apoptotic regulation [64, 65]. Progression of NMIBC to higher-grade muscle invasive disease is also due to alterations in TP53 and RB1. Early studies by Sarkis et al. [66, 67] found TP53 alterations to be strong independent predictors of disease progression in patients with NMIBC, MIBC, and CIS. Recent studies have supported these findings by showing an independent role of TP53 alteration in predicting disease-free survival and disease-specific survival in patients with pT1 and pT2 tumors who have undergone cystectomy [68].

Digital quantitative detection of nuclear p53 by immunofluorescence staining of histological samples seems to provide more objective and reproducible values corresponding to p53 protein concentration in cell's nuclei than the traditional scoring system of counting the positively stained cells [69].

As it has been proved in previous publications of our working group [9–13] quantitative expression analysis of these proteins seems to be helpful to establish prognosis in BC.

## 4. Population Investigated

### 4.1. Clinical Methodology

This analysis used a subpopulation of a previous clinical database with three different groups of patients, NMIBC, MIBC, and Benign Prostatic Hyperplasia (BPH) patients. 45 patients with a new diagnosis of NMIBC were selected. Patients with BPH, MIBC, CIS, and previous NMIBC recurrent tumors were not included in this database because of their different clinical behavior.

Anamnesis and physical examination with clinical history were previously carried out in order to collect clinical factors (age, sex, smoking status, and alcohol consumption and presentation mode).

As part of a preoperative staging, preoperative imaging (renal and bladder ultrasound, intravenous urography, computed tomography, or cystoscopy) and urine cytology were carried out before the diagnosis of all patients.

After that, patients underwent conventional TURBT and the following data were collected: multiplicity, size, and aspect. TURBT was completed with a standardized multiple biopsy of the bladder surface in order to exclude the presence of concomitant CIS.

Once the TURBT was finished, the tumor tissue obtained was divided into two specimens: one of them for the histopathological study and the other one for protein expression studies.

Histopathological diagnosis was performed by a single pathologist. Grading was established using the OMS classification [70]. Staging was performed by the UICC criteria 1997 staging system [71]. Patients with biopsies that showed the presence of concomitant CIS were excluded from the study.

The samples extracted in the surgery room were sent to the proteomic laboratory for a quantification of the following biomarkers:

- *p53* protein: quantified in the cytosol by a technique of immunoluminescence (LIA);
- *neu* protein: determined using a quantitative enzyme linked immunoassay (ELISA);
- *EGFR*: quantified in membranes by radioimmunoassay (RIA).

Then, a stratified protocol of postoperative adjuvant intravesical therapy and standard follow-up for patients diagnosed NMIBC with cytology and cystoscopy or ultrasound was performed for preventing and detecting tumor recurrence and/or progression.

### 4.2. Dataset

The dataset used in the experiments is composed of 45 patients undergoing TURBT for NMIBC without the presence of concomitant CIS.  Table 1 summarizes the 67 variables measured for each patient, their description, and range.

## 5. Hierarchical Clustering Analysis

The numerical analyses performed here with the dataset emphasized the use of clustering algorithms for finding hierarchical groups of objects in an unsupervised way [72–74]. The first steps involved preparing the dataset for analysis, which included cleansing and normalizing the data. Then, three different clustering analyses were performed: using only those variables with no missing values; using all variables, but replacing missing values; and using only those variables selected by experts. The different analyses allowed us to detect, remove, and explain anomalies in the dataset and to cluster patients based on* neu* ranges and risk groups, with a different prognostic of progression or recurrence. The method and experiments are detailed in the following sections.

### 5.1. Single-Linkage Hierarchical Clustering

Clustering, in data mining, tries to identify the distribution of patterns and intrinsic correlations in datasets by partitioning the data points into similarity groups. Clustering enhances the value of existing databases by revealing rules in the data. These rules are useful for understanding trends, making predictions of future events from historical data, or synthesizing data records into meaningful clusters [72–74].

Clustering algorithms usually employ a distance metric (e.g.,* Euclidean*) or a similarity measure to partition the database, such that data points in the same partition are more similar than points in different partitions. Hierarchical clustering is one of the most frequently used methods in unsupervised learning. Given a set of data points, the output is an upside down tree, known as a dendrogram, whose leaves are the data points and whose internal nodes represent nested clusters of various sizes. The tree organizes these clusters hierarchically, where the hope is that this hierarchy agrees with the intuitive organization of real-world data.

The method used in the clustering experiments performed in this paper is named single-linkage. This is an agglomerative hierarchical method in which new clusters are created by combining the most similar groups. The initial clustering is formed by a singleton, that is, a single object, and at each iteration a new cluster is formed by joining two of the most similar groups of the previous iterations. In the single-linkage, the distance between the new group and the others is determined as the shortest distance among the elements of the new and the remaining groups.

### 5.2. Data Cleansing

Data preprocessing, or data preparation, manipulates and transforms data so that the knowledge contained in it can be more easily and accurately extracted [75, 76]. The best way to preprocess the data depends on three main issues: the database problems (e.g., inconsistency and noise); what use is intended from the data; and how the data analysis tools to be used work.

The first preprocessing step performed with the dataset was to remove constant-valued variables, identifiers (IDs), variables with a high number of missing values, and dates.  Table 2 presents the variables that were removed from the original dataset and why.

### 5.3. Analysis with No Missing Values

In this first analysis, only those variables without missing values were considered, totaling 58 out of 67 variables, as follows: Age; Gender; Tabaco; Alcohol; Af; Mfumador; Hematuri; Irritat; Dolorsup; Otross; Diagn; Tumor; Hem; Hb; Hcto; Citesp; Citar; Eco; Uiv; Ct; Cistosc; Multiple; Tam; TAM3CM; Aspect; ASPESUPE; Tto; ADYUV; Oncot; Mytom; Bcg; Bmn; Jewett; G; G23; Tnm; GRIES; GRX; Ap; Tipoap; p53iha; p53ria; Neu; Recidiv; Progres; Nrecidiv; Numrecp; Metas; Muerte; Mporca; Recm; Logneu; Superv; Tprogre; Tmetas; Np53ria; Nneu; Edad70.

**Figure 1:**
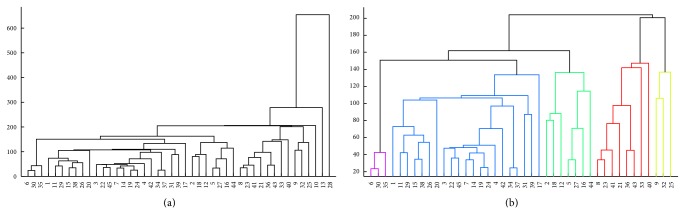
Hierarchical clustering of the NMIBC patients removing variables with missing values. (a) Clustering of the whole dataset. (b) Clustering of the dataset after removing the anomalous patients 10, 13, and 28.

Figure 1(a) shows the dendrogram of the hierarchical clustering performed on all patients and only those variables with no missing values. It can be observed that patients 10, 13, and 28 have profiles substantially distinct from the others, thus being treated as anomalies. To better investigate the data and search for groups of patients' profiles, the anomalous patients (10, 13, and 28) were removed from the dataset and a new hierarchical clustering was performed, as depicted in Figure 1(b).

By looking at Figure 1(b) the presence of five clusters of patients can be observed (represented here by their IDs):

- *Cluster 1*: 6, 30, 35;
- *Cluster 2*: 1, 3, 4, 7, 11, 14, 15, 17, 19, 20, 22, 24, 26, 29, 31, 34, 37, 38, 39, 42, 45;
- *Cluster 3*: 2, 5, 12, 16, 18, 27, 44;
- *Cluster 4*: 8, 21, 23, 33, 36, 40, 41, 43;
- *Cluster 5*: 9, 25, 32.

After an analysis of the original dataset and comparison with the groups found by the algorithm, it is possible to note a subdivision of patients based on ranges of the* neu* variable, as follows:

- *Cluster 1*: neu ≈ 400 HNU/mL;
- *Cluster 2*: 600 ≤* neu* ≤ 1,100 HNU/mL;
- *Cluster 3*:* neu* < 400 HNU/mL;
- *Cluster 4*: 1,500 ≤* neu* ≤ 1,900 HNU/mL;
- *Cluster 5*: 1,200 ≤* neu* ≤ 1,400 HNU/mL.

The anomalous profiles found presented a very large* neu*:* neu* > 1,900 HNU/mL. No association between these* neu* clusters and classical risk factors or risk groups was found.

### 5.4. Analysis Replacing Missing Values

In the second set of experiments performed, all 67 variables were used, but those with missing values were replaced by the average in case of numeric variables, or by the mode in case of categorical variables.  Table 3 summarizes the replacement values used for each variable with missing values.

**Figure 2:**
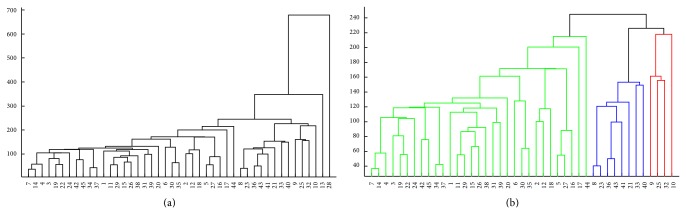
Hierarchical clustering of the NMIBC patients using all variables. (a) Clustering of the whole dataset. (b) Clustering of the dataset after removing the anomalous patients 13 and 28.

In this case, the hierarchical clustering shown in Figure 2(a) indicates only two anomalous profiles, patients 13 and 28. After removing them from the dataset, the following clusters emerge (Figure 2(b)):

- *Cluster 1*: 1, 2, 3, 4, 5, 6, 7, 11, 12, 14, 15, 16, 17, 18, 19, 20, 22, 24, 26, 27, 29, 30, 31, 34, 35, 37, 38, 39, 42, 44, 45;
- *Cluster 2*: 8, 21, 23, 33, 36, 40, 41, 43;
- *Cluster 3*: 9, 10, 25, 32.

In this case, an analysis of the groups formed leads to the observation of the following* neu* ranges: Cluster 1 (1,250 HNU/mL ≤* neu* ≤ 1,550 HNU/mL); Cluster 2 (*neu* > 1,550 HNU/mL); and Cluster 3 (*neu* < 1,200 HNU/mL).

No association between these* neu* clusters and classical risk factors or risk groups was found.

### 5.5. Expert Selection of Relevant Variables

**Figure 3:**
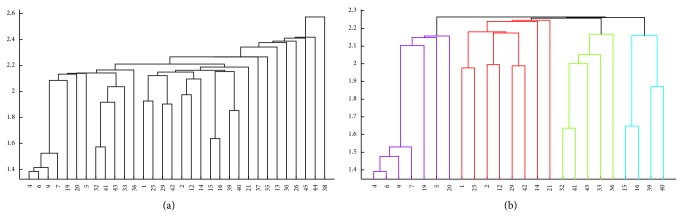
Hierarchical clustering of the NMIBC patients using the selected variables. (a) Clustering of the whole dataset. (b) Clustering of the dataset after removing patients 13, 26, 30, 35, 37, 38, 44, and 45.

In this last experiment, the goal was to observe if there is any relationship between the molecular markers (proteins* neu*, EGFR, and p53) and the tumoral tissue of NMIBC. To investigate that, a subset of the variables was selected manually and the clustering algorithm was applied. The following variables were chosen: Age, Gender, Tabaco, Tumor, Multiple, Tam, TAM3CM, ASPESUPE, G, G23, Tnm, GRIES, GRX, Tipoap, p53iha, p53ria, neu, Recidiv, Progres, Nrecidiv, Metas, Muerte, Egfr, Logneu, Superv, Ile, Tprogre, Tmetas, Np53ria, Nneu, Negfr, and Edad70. The patients with missing values (3, 8, 10, 11, 17, 18, 22, 23, 24, 27, 28, 31, and 34) were removed from the dataset. The results are presented in Figure 3. In Figure 3(a) the clustering of the whole dataset is presented, and the presence of eight outliers can be observed: 13, 26, 30, 35, 37, 38, 44, and 45.

By looking at Figure 3(b) it is possible to observe four clusters of patients who can be subdivided into risk groups:

- *Cluster 1*: low risk with no progression and recurrence;
- *Cluster 2*: high risk with no recurrence (or late recurrence) and no progression;
- *Cluster 3*: low risk with early recurrence and no progression;
- *Cluster 4*: high risk with recurrence and progression.

Outlier patients can always be grouped in one of the different clusters according to their clinical characters (size, number, grade, stage, etc.) but were excluded by the algorithm because one or more molecular markers were out of range, as shown in Tables 4 and 5.

### 5.6. Discussion

Progress in data storage and acquisition has resulted in a growing number of enormous databases. The information contained in these databases can be extremely interesting and useful; however, the amount is too large for humans to process manually. Data mining is defined as part of knowledge discovery in databases and draws on the fields of statistics, machine learning, pattern recognition, and database management and can be able to extract interesting and useful material from these large datasets.

Using a hierarchical algorithm it was possible to find two different cluster associations based on HER2/neu levels. None of these associations was significantly correlated with any of the clinicopathologic data studied (neither classical risk factors nor risk groups). These data support the previous assertion of another working group, which suggested that the quantitative assessment of HER2/neu expression by ELISA in BC was not significantly associated with stage or grade and has no prognostic significance by itself but only aided by other proliferation markers such as SPF, DI, and ploidy [54].

By using a hierarchical clustering algorithm, an interesting distribution of patients into four different groups (clusters) with different biological behaviors and prognosis could be found. Cluster 1 is composed of unique tumors, low size (<3 cm), low grade, and low stage, with a low risk of relapse or progression, and with a biological behavior according to the expected one in patients with these characteristics. Cluster 2 is composed of tumors with a high risk of relapse and progression (multiplicity, bigger size than 3 cm, high grade, and high stage) but with no relapse (or a very late superficial relapse) and no evidence of progression during a long follow-up period (almost 8 years). Cluster 3 is composed of unique tumors, with low size, low grade, low stage, and a low risk of relapse or progression, that shows a very early relapse as NMIBC and no progression. Cluster 4 is composed of high risk tumors, with a high risk of progression (multiplicity, bigger size, high grade, and high stage) and with a biological behavior according to these characteristics, with an early relapse, progressing to a MIBC.

Outlier patients can always be grouped into one of the different clusters according to their clinical characters (size, number, grade, stage, etc.) and biological behavior but were excluded by the algorithm because one or more molecular markers were out of range. Nevertheless, no rules of distribution between clusters and any of the molecular markers were found.

The small number of patients in the database due to the restrictive criteria of inclusion (NMIBC, first tumor, no CIS associated, and disposable molecular markers) and the retrospective analysis of a preexisted database with no specific design for this use were important limitations of the present study.

## 6. Conclusions and Future Work

This paper explored the hypothesis that clinical and histopathological data, together with information from several molecular markers in patients, helps in the prediction of outcomes and design of treatments for nonmuscle invasive bladder cancer. A hierarchical clustering algorithm was applied to a set of patients to identify clusters of patients with clinical, molecular markers and prognostic factors and provide statistics about the recurrence, progression, and survival of patients.

The results presented showed that the cluster algorithms can group patients with NMIBC into different molecular clusters. The quantitative assessment of HER2/neu expression in NMIBC was grouped by the algorithm, but these were not significantly correlated with clinicopathologic data and are not useful for predicting the patients' outcome. Also, EGFR and p53 showed not to be useful proteins for clustering patients with NMIBC. However, the hierarchical clustering algorithm could group patients with NMIBC into different risk groups with different clinical behaviors and prognosis, but these ones were not significantly correlated with molecular markers. Outliers were also detected and explained.

Future investigation includes the use of a larger number of patients and the inclusion of different molecular markers in the analyses.

## Figures and Tables

**Table 1:** Variables measured and available in the dataset.

Name	Description	Values	Significance
Type	Type of sample	1/2/3	NMIBC/MIBC/control
Age	Diagnosis age	Numeric	Years
N History	Identification number	Numeric	—
Gender	Gender	1/2	Male/female
Fdiagn	Diagnostic data	Date	DD/MM/YYYY
Tobacco	Tobacco smoking	0/1	No/yes
Alcohol	Alcohol consumption	0/1	No/yes
Af	Family history of BC	0/1	No/yes
Mfum	More than 20 cigarettes a day	0/1	No/yes
Otrosf	Other risk factors of BC	Text	Not analyzable
Hematuri	Haematuria	0/1	No/yes
Irritat	Irritative syndrome	0/1	No/yes
Dolorsup	Suprapubic pain	0/1	No/yes
Otros	Other symptoms	0/1	No/yes
Diagn	Diagnostic type	1/2	Symptomatic/incidental
Tumor	Number of tumors	Numeric	Numeric
Creat	Creatinine	Numeric	mg/dL
Got	GOT	Numeric	U/L
Gpt	GPT	Numeric	U/L
Hem	Number of red blood cells	Numeric	E^6^/uL
Hb	Haemoglobin	Numeric	g/dL
Hcto	Hematocrit	Numeric	%
Ca	Calcium	Numeric	mg/dL
P	Phosphorum	Numeric	mg/dL
Falc	Alcaline phosphatase	Numeric	U/L
Citesp, Citarr; eco, UIV; CT, cistosc	Diagnosis test performed	Text	Not analyzable
Multiple	Multiplicity	1/2	Single/multiple
Tam	Size (cm)	numeric	cm
TAM3CM	Size ≥3 cm	1/2	No/yes
Aspect	Endoscopic aspect	1/2/3	1 Superficial/2 infiltrative/3 intermediate
ASPESUP	Superficial aspect	1/2	Yes/no
Tto	Type of adjuvant therapy	Text	Not analyzable
ADYUV	Adjuvant therapy	1/2	Yes/no
Jewett	Histologic staging	1/2/3	A/B/C-D
G	Grade	1/2/3	G1/G2/G3
G23	Grades 2 or 3	1/2	No/yes
Tnm	TNM	1/2	Ta/T1
Gries	EORTC risk group	1/2	Low-intermediate/high
Grx	Millan risk group	1/2	Low-intermediate/high
AP, tipoAP	Type of BC	1/2/9	Not analyzable TCC/SC/other
p53iha	P53 immunohistochemistry	1/2/3	+/++/+++
p53ria	P53 quantified	Numeric	ng/ml
Neu	Prot p185 quantified	Numeric	HNU (0.05 fmol/mg)/ml
p16	Prot p16 immunohistochemistry	1/2/3	+/++/+++
Recid	Relapse	1/2	Yes/no
Fechare	First relapse data	Date	DD/MM/YYYY
n^a^recid	Number of relapses	Numeric	Number
n^a^recidp	Number of relapses till progression	Numeric	Number
Prog	Progression	1/2	Yes/no
Fprog	Progression date	Date	DD/MM/YYYY
Metas	Metastatic disease	1/2	Yes/no
Muerte	Death	1/2	Yes/no
Fechmuerte	Date of death	Date	DD/MM/YYYY
Mporca	Cancer specific mortality	1/2	Yes/no
Recm	Number of relapses till death	Numeric	Number
fechaultre	Last Revision Date	Date	DD/MM/YYYY
Egfr	EGFR quantified	Numeric	EGFR fmol/protein mg
Logneu	Neu logarithm	Numeric	Number of months
Super	Survival (months)	Numeric	Number of months
Ile	Relapse-free survival	Months	Number of months
Tprogre	Progression-free survival	Months	Number of months
Tmetas	Metastatic disease-free survival	Months	Number of months
Np53ria	p53 RIATertile	1/2/3	Tertile 1/tertile 2/tertile 3
Nneu	NeuTertile	1/2/3	Tertile 1/tertile 2/tertile 3
Negfr	EGFRTertile	1/2/3	Tertile 1/tertile 2/tertile 3
Filtro	NMIBC		
edad70	Older than 70 years	1/2	Yes/no

**Table 2:** Variables removed from the dataset during the cleansing process.

Variable	Explanation
Type	Constant value
Nhistori	Identifier
Otrosf	87% of missing values (additional medical information)
Creat	Empty
p16	Empty
Otrosm	93% of missing values (additional medical information)
Fdiagn	Date
Fecharec	Date
Fechaprog	Date
Fechametas	Date
Fechmuerte	Date
Fechaultre	Date
filter_$	Constant value

**Table 3:** Replacement values for the variables with missing values.

Variable	Value
Got	25
Gpt	24
Ca	9.32
P	3.22
Falc	87
h_c	23
Egfr	10.05
Ile	61
Negfr	3

**Table 4:** Clinical and molecular characteristics of the different clusters.

Attribute	Cluster 1	Cluster 2	Cluster 3	Cluster 4
Age^*^	61 years; 18	67 years; 9	70 years; 9	82 years; 9
	(23–73)	(52–79)	(60–82)	(72–93)

Multiplicity	No: 100%	No: 20%	No: 40%	No: 50%
	Yes 0%	Yes 80%	Yes 60%	Yes 50%

TM > 3 cm	No: 71%	No: 50%	No: 60%	No: 0%
	Yes: 29%	Yes: 50%	Yes: 40%	Yes: 100%

Grade	G1: 83.3%	G1: 0%	G1: 20%	G1: 0%
	G2: 16.6%	G2: 62.5%	G2: 80%	G2: 50%
	G3: 0%	G3: 37.5%	G3: 0%	G3: 50%

TNM	Ta: 100%	Ta: 0%	Ta: 80%	Ta: 0%
	T1: 0%	T1: 100%	T1: 20%	T1: 100%

Risk group [26]	Low-Int: 100%	Low-Int: 0%	Low-Int: 80%	Low-Int: 0%
	High: 0%	High: 100%	High: 20%	High: 100%

Risk group [38]	Low-Int: 100%	Low-Int: 12.5%	Low-Int: 100%	Low-Int: 0%
	High: 0%	High: 87.5%	High: 20%	High: 100%

p53 (ng/mL)^*^	0.1; 0.2	0.5; 1.2	0; 0	0; 0
	(0–0.6)	(0–3.40)	(0-0)	(0-0)

neu (HNU/mL)^*^	748.5; 415.6	775.4; 544.2	1379.9; 184.7	854.4; 497.7
	(328–1596.1)	(76–1749.1)	(1253.0–1698.1)	(330.9–1527.2)

EGRF (fmol/mg)^*^	6.9; 4.0	12.5; 12.8	8.5; 4.7	22.8; 17.2
	(0.2–11.4)	(2.2–16.6)	(3.0–15.1)	(7.1–39.5)

GS (months)^*^	104; 37 (47–128)	93; 46 (3–135)	84; 47 (23–133)	17; 10 (5–28)

RFS (months)^*^	104; 37 (47–128)	81; 56 (3–135)	9; 4 (4–13)	13; 10 (5–27)

PFS (months)^*^	104; 37 (47–128)	93; 46 (3–135)	84; 47 (23–133)	13; 10 (5–27)

^*^Mean; SD (range).

**Table 5:** Clinical and molecular characteristics of the different outliers (patient number).

Attribute	Outlier 1 (13)	Outlier 2 (26)	Outlier 3 (30)	Outlier 4 (35)	Outlier 5 (37)	Outlier 6 (38)	Outlier 7 (44)	Outlier 8 (45)
Age	77	73	69	72	41	71	80	60
Multiplicity	Yes	Yes	Yes	No	No	No	No	Yes
TM > 3 cm	Yes	No	No	No	No	No	Yes	Yes
Grade	G3	G2	G2	G2	G2	G2	G3	G2
TNM	T1	Ta	Ta	Ta	T1	T1	T1	Ta
Risk group	High	Low-Int	Low-Int	Low-Int	High	High	High	Low-Int
p53	0	**4.7**	**0.9**	0	**0**	0	**0.9**	0
Neu	**2,125.80**	**724.5**	**415.8**	**459**	994	700	385.7	**870.6**
Egfr	**0.5**	6.3	**15.5**	8.6	3.2	**1.3**	16.4	**0**
GS (months)	15	35	133	111	131	11	9	106
RFS (months)	15	11	5	11	59	1	3	18
PFS (months)	15	35	133	111	131	1	3	91

Cluster	4	3	3	3	2	4	4	3

**Bold: **reason for exclusion by the algorithm.
